# Associations of childhood maltreatment types and timing with oxidative stress in formerly out-of-home placed young adults

**DOI:** 10.1080/20008066.2026.2711481

**Published:** 2026-09-02

**Authors:** Clara Von Wendorff, Maria Meier, Inga Schalinski, Daniella Dwir, Basilio Giangreco, Paul Klauser, Katharina Beck, Gizem Demirkiran, Patricia Schneider, Mickaël Das Neves, Cyril Boonmann, Marc Schmid, Jörg M. Fegert, David Bürgin, Vera Clemens

**Affiliations:** a Child and Adolescent Psychiatry, Psychosomatics, and Psychotherapy, University Hospital Ulm, Ulm, Germany; b Research Department Clinic for Child and Adolescents, University Psychiatric Hospital Basel, Basel, Switzerland; c Faculty of Human Sciences, Institute of Psychology, University of the Federal Armed Forces Munich, Neubiberg, Germany; d Center for Psychiatric Neuroscience, Psychosis Research Unit, University of Lausanne, Prilly, Switzerland

**Keywords:** Oxidative stress, redox dysregulation, childhood adversity, childhood maltreatment, abuse, neglect, timing, duration, care leavers, mental health, Estrés oxidativo, desregulación redox, adversidades en la infancia, maltrato infantil, momento en que se producen, períodos de vulnerabilidad, duración, salud mental

## Abstract

**Background:** Childhood maltreatment (CM) is associated with an increased risk of mental disorders, with oxidative stress (OS) being proposed as a potential mediator. However, it remains unclear whether exposure to specific types of CM during distinct developmental periods is associated with altered OS levels.

**Objective:** This study aimed to explore the association between CM type and timing and OS in young adulthood.

**Methods:** We studied 116 adults (30.5% women; mean age = 26.3 years) with previous residential care placements. CM exposure was assessed with the Maltreatment and Abuse Chronology of Exposure (MACE-X) scale, capturing types (parental abuse, neglect, peer violence, sexual abuse), overall severity, multiplicity, and duration. OS was measured via blood-based biomarkers: superoxide dismutase (SOD), glutathione peroxidase (GPx), glutathione reductase (GRed), and GPx/GRed ratio. Conditional random forest regressions and Spearman correlations were used to test effects of CM types and timing on OS.

**Results:** Abuse and neglect during adolescence showed the highest variable importance (VI) for GRed. Abuse was associated with increased, and neglect with decreased GRed. Neglect at ages 8 and 12 showed the highest VI for GPx/GRed and were positively associated with the biomarker ratio. Furthermore, no significant associations were found between overall CM severity, multiplicity, or duration and any of the OS biomarkers examined.

**Conclusion:** Type- and timing-specific characteristics may be important when examining associations between CM and OS in adulthood. More research is needed to test whether such associations can inform preventive strategies that aim to mitigate mental health impairments following CM.

## HIGHLIGHTS

- The findings indicate sensitive periods in childhood and adolescence during which exposure to parental abuse and neglect is associated with alterations in oxidative stress (OS) in young adulthood.
- Neglect during middle childhood and adolescence was associated with lower levels of glutathione reductase (GRed), whereas abuse during adolescence was associated with higher GRed.
- Overall severity of childhood maltreatment and cannabis use emerged as the predictors with the highest variable importance for glutathione peroxidase (GPx). However, neither predictor showed a statistically significant association with GPx levels.
- Higher levels of neglect at ages 8 and 12 and medication use were associated with an increased GPx/GRed ratio.
- The cumulative duration of childhood maltreatment was not associated with any OS marker.

## Abstract (es)

**Antecedentes:** El maltrato infantil (MI) se asocia con un mayor riesgo de trastornos mentales, y se ha propuesto que el estrés oxidativo (EO) podría ser un posible mecanismo mediador. Sin embargo, aún no está claro si la exposición a tipos específicos de MI durante distintos periodos del desarrollo se asocia con alteraciones en los niveles de EO.

**Objetivo:** El objetivo de este estudio fue explorar la asociación entre los tipos y el momento de exposición al maltrato infantil y el EO en la adultez temprana.

**Métodos:** Se estudió a 116 adultos (30,5% mujeres; edad media = 26,3 años) que habían estado bajo acogida residencial. La exposición al maltrato infantil se evaluó mediante la escala de Cronología de la Exposición al Maltrato y Abuso (MACE por sus siglas en ingles), que recoge los tipos (maltrato parental, negligencia, violencia entre pares, abuso sexual), la gravedad general, la multiplicidad y la duración. La salud general se midió mediante múltiples biomarcadores sanguíneos: superóxido dismutasa (SOD), glutatión peroxidasa (GPx), glutatión reductasa (GRed) y la relación GPx/GRed. Se utilizaron regresiones de bosque aleatorio condicional y correlaciones de Spearman para evaluar los efectos de los tipos y el momento del maltrato infantil sobre el EO.

**Resultados:** El maltrato y el abandono durante la adolescencia fueron los predictores con mayor importancia de la variable (VI) para la GRed. El abuso se asoció con un aumento de la GRed, mientras que el abandono se asoció con una disminución de esta. La negligencia a los 8 y a los 12 años fueron los predictores con mayor VI para GPx/GRed y se asociaron positivamente con la relación de biomarcadores. Además, no se encontraron asociaciones significativas entre la gravedad general, la multiplicidad o la duración del maltrato infantil y ninguno de los biomarcadores EO examinados.

**Conclusión:** Las características específicas del tipo y el momento en que se produjo el maltrato infantil pueden ser factores importantes a la hora de examinar los vínculos entre el maltrato infantil y el EO en la edad adulta. Se necesitan más investigaciones para comprobar si dicha asociación puede servir de base para estrategias preventivas destinadas a mitigar los trastornos de salud mental derivados del maltrato infantil.

## 1. Introduction

### 1.1. Childhood maltreatment and dysregulation in physiological systems

Childhood maltreatment (CM), including parental abuse and neglect, has profound and lasting consequences for both physical (Grummitt et al., 2021; Hughes et al., 2017) and mental health (Baldwin et al., 2023; Xiao et al., 2023). Individuals with a history of CM exhibit an increased risk for psychiatric disorders such as anxiety disorders, psychosis, depression, and post-traumatic stress disorder (Baldwin et al., 2023; McKay et al., 2022; Teicher et al., 2022). Emerging evidence suggests that CM is associated with dysregulation across multiple physiological systems, including the nervous, metabolic, inflammatory, and endocrine system. It has been proposed that these dysregulations contribute to the heightened risk of psychopathology following CM (Chiang et al., 2022; Hakamata et al., 2022; Mulkey & du Plessis, 2019; Nousen et al., 2014). Despite these well-established associations, the underlying biological mechanisms remain insufficiently understood.

### 1.2. The relationship between childhood maltreatment and oxidative stress

A promising avenue for further investigation is the role of oxidative stress (OS). OS refers to an imbalance between prooxidant molecules, such as reactive oxygen species (ROS), and antioxidant defences, such as glutathione (GSH) and superoxide dismutase (SOD), and is closely linked to inflammation (Biswas, 2016; Lugrin et al., 2014). The increase in prooxidants and decrease in antioxidants associated with OS may lead to cellular damage, including the oxidation of DNA and RNA, protein carbonylation, lipid peroxidation (Valko et al., 2007), and telomere shortening (Epel & Prather, 2018). OS can therefore be conceptualized as a manifestation of a broader disruption in redox homeostasis. Notably, OS has been implicated in various psychiatric conditions (Jorgensen et al., 2022), including psychosis (O’Donnell et al., 2014), post-traumatic stress disorder (PTSD) (Miller et al., 2018; Oroian et al., 2021), depression (Balmus et al., 2016; Lievanos-Ruiz & Fenton-Navarro, 2024), bipolar disorder (Jiménez-Fernández et al., 2021), and anxiety disorders (Fedoce et al., 2018; Maes et al., 2018) and has thereby been discussed as a potential mechanism that increases the risk for diseases.

A growing body of evidence suggests that CM is associated with elevated OS levels. Such associations have been reported in healthy adolescents (do Prado et al., 2016), adolescence with major depressive disorder (Chen et al., 2024), adults with affective disorders and their healthy relatives (Moraes et al., 2018), and in postpartum women (Boeck et al., 2019). However, findings remain heterogeneous, with some studies reporting increased OS levels, others reporting decreased levels, and still others finding no significant differences (Karanikas et al., 2021; von Wendorff et al., 2026). These studies mostly relied on aggregated CM measures that cumulate the severity of exposure across childhood and adolescence. As such, they did not consider potential differences in CM type, timing, and duration – variables that have been shown to differentially shape the risk of psychopathology (Juen et al., 2024; Meier, Schalinski, et al., 2025; Schalinski et al., 2016) as well as neurobiology following CM. For example, associations between the timing and duration of CM and structural brain alterations, including changes in the anterior cingulate cortex (Grauduszus et al., 2024), amygdala and hippocampus volume (Pechtel et al., 2014; Sicorello et al., 2021), and cortical thickness (Türkmen et al., 2024) have been reported in the past. In addition, maltreatment during early childhood was associated with epigenetic aging in later childhood and adolescence (Meier, Gurri, et al., 2025). These findings underscore the intricate interplay between developmental timing, CM types, and region-specific brain alterations (Gee, 2021). On a molecular level, CM before the age of 5 in girls has been linked to elevated inflammation levels, whereas prolonged CM exposure has been associated with persistently increased inflammatory markers (Ehrlich et al., 2021). Findings from animal models furthermore indicate that CM during the peripubertal period may alter OS levels and increase anxiety-related behaviours (Schnider et al., 2022). Taken together, the evidence that CM type, timing, and duration differentially affect neuroanatomical and molecular processes suggests that these dimensions may likewise influence OS, highlighting the need for a more nuanced investigation of their role in shaping long-term psychological vulnerability following CM.

### 1.3. Type- and timing-specific characteristics of childhood maltreatment and mental health

Beyond alterations in neurobiology, the type, timing, and duration of CM also exert a substantial influence on mental health outcomes. For example, the timing of CM appears to shape risk profiles for clinical outcomes. Adversities at age 9 predicted youth psychopathology more strongly than those at earlier ages, except for neglect, which was most predictive of internalizing symptoms when occurring at age 3 (Hardi et al., 2024). Exposure during middle childhood (6–13 years) carried the highest risk for depression, followed by late childhood (12–19 years) and early childhood (0–6 years) (Li et al., 2023). Further, distinct types of CM that are experienced at particular developmental stages confer differential risks: sexual abuse before age 5 and physical abuse after age 13 are especially predictive of adult depression and PTSD symptoms (Capretto, 2020). In formerly out-of-home placed young adults, parental abuse in early to middle childhood, peer violence during adolescence, and sexual abuse at age 18 were associated with overall mental health problems. In the same cohort, internalizing problems were linked to parental abuse at age 6, sexual abuse at ages 17–18, and adolescent peer violence, while externalizing problems were associated with parental abuse in early to middle childhood, peer violence during adolescence, and sexual abuse at age 18 (Meier, Gurri, et al., 2025). Collectively, these findings suggest that CM is associated with differential psychopathological outcomes dependent on its type and timing (Russotti et al., 2021), and no consistent or universally accepted vulnerable period has been identified (Schaefer et al., 2022). Taken together, the literature highlights the need to jointly consider CM type, timing, and duration when assessing long-term neurodevelopmental, physiological, and mental health consequences.

### 1.4. Research question and study hypotheses

While existing research indicates that CM characteristics, such as type, timing, and duration, contribute to its neurobiological and mental health consequences, studies specifically examining their associations with OS remain limited. We therefore conducted competitive hypothesis testing to compare the relative importance of CM type, timing, and global indicators of CM exposure. We hypothesized that CM type and timing would be more strongly associated with OS biomarkers than global indicators and would therefore demonstrate greater explanatory value.

Given the limited evidence regarding specific associations between CM characteristics and OS, the present analysis was otherwise exploratory, and no a priori hypotheses were formulated regarding which CM types or developmental periods would show the strongest associations with OS in adulthood. We aimed to examine the extent to which CM type, timing, duration, multiplicity, and overall severity were associated with OS biomarkers in a sample of young adults with histories of residential care placement. To account for the simultaneous influence and potential interrelations of all predictors, we applied conditional random forest regression analyses to identify the CM characteristics most strongly associated with OS in young adulthood. Subsequently, regression models and t-tests were conducted to further characterize the direction of these associations.

## 2. Method

### 2.1. Sample and procedure

This study used data from the Swiss Study for ‘Clarification and Goal-Attainment in Child Welfare and Juvenile Justice Institutions’ (MAZ., 2007–2012), which included 592 individuals (aged 5–27, mean = 15.86, *SD* = 2.99, 32.1% female) from 64 Swiss residential care institutions. In the course of the follow-up study ‘Child Welfare Trajectories: Learning from Experience’ (JAEL), 511 participants who had agreed to be recontacted were attempted to be reached again, with 231 completing online questionnaires and 185 participating in face-to-face assessments between 2018 and 2020 (mean age = 26.28, *SD* = 3.63, range = 16–38, 30% female). A total of 131 participants provided a blood sample, of whom 116 had available OS biomarker data (see flowchart in the Supplementary Material). The sample size of the present study is comparable to those reported in previous investigations of clinical and high-risk populations using the MACE-X questionnaire in combination with conditional random forest regression models, which ranged from 43 to 341 participants (Fujisawa et al., 2018; Grauduszus et al., 2024; Hutson et al., 2024; Juen et al., 2024; Meier, Gurri, et al., 2025; Pechtel et al., 2014; Schalinski et al., 2016; Schalinski et al., 2018; Schalinski et al., 2019; Schalinski & Teicher, 2015; Sicorello et al., 2021). Approximately half of these studies included larger samples and half included smaller samples than the present study. All participants gave written informed consent and received supermarket vouchers valued up to CHF 400 for taking part in the JAEL study, plus an additional CHF 100 for blood collection. The study adhered to the Declaration of Helsinki and was approved by the Ethics Commission of North-western and Central Switzerland. Prior analyses of the dataset examined associations between CM and outcomes such as psychopathology (Meier, Gurri, et al., 2025), personality functioning (d’Huart et al., 2022), telomere length, hair cortisol (Bürgin et al., 2022), epigenetic aging (Meier et al., 2024), and OS and mental health (von Wendorff et al., 2026).

### 2.2. Sociodemographic characteristics

A computerized self-report questionnaire was administered to collect sociodemographic data, including age at blood draw, age at first placement, total number of placements, nationality, and sex (self-reported sex assigned at birth). Potential confounders were also assessed and included smoking status, fasting status at the time of blood draw, body mass index (BMI), substance use within the past 30 days (cannabis, cocaine, opiates, amphetamines), current medication use, and the time interval between blood collection and laboratory processing. To descriptively assess the prevalence of mental disorders, the Structured Clinical Interview for DSM-IV (SCID-I and SCID-II) was administered (First & Gibbon, 2004). Risky alcohol consumption was assessed using the Alcohol Use Disorders Identification Test (AUDIT) (Saunders et al., 1993).

### 2.3. Childhood maltreatment

CM was assessed using the Maltreatment and Abuse Chronology of Exposure scale (MACE-X) (Teicher & Parigger, 2015), a 75-item questionnaire administered during the face-to-face session. Participants reported if and when (1–18 years of age) they experienced specific events (e.g. being insulted by parents/caregivers). The MACE-X scale differentiates between ten types of CM: parental physical abuse, parental verbal abuse, parental non-verbal emotional abuse, sexual abuse, witnessing violence toward parents, witnessing violence toward siblings, peer emotional violence, peer physical violence, emotional neglect, and physical neglect. To reduce the large number of predictors in subsequent analyses, these types were grouped into four categories: *ABUSE* (parental abuse and witnessed violence), *NEGLECT* (emotional and physical neglect), *PEER* (peer-related violence), and *SEXA* (sexual abuse) according to previous studies (Meier, Gurri, et al., 2025; Schalinski et al., 2019). Severity scores for each category were calculated for each age from 3 to 18 years, ranging from 0–10, by averaging and weighting subscale scores. Additionally, three global CM measures were derived: *DURATION* (years of exposure to any CM above a predefined threshold; Teicher & Parigger, 2015), *SEVERITY* (overall CM severity across all CM types), and *MULTI* (number of CM types experienced above the threshold). These global measures and the time and subtype specific variables served as predictors in conditional random forest regressions. Reports from ages 1–2 were excluded due to low reliability, consistent with prior research (Cordon et al., 2004; Meier, Gurri, et al., 2025; Sicorello et al., 2021).

### 2.4. Oxidative stress markers

*Superoxide dismutase (SOD)* activity facilitates the conversion of superoxide radicals into hydrogen peroxide. Superoxide radicals are generated primarily through the action of xanthine oxidase, an enzyme whose activity is suppressed by SOD. Therefore, SOD’s ability to inhibit xanthine oxidase can be evaluated by quantifying the build-up of superoxide radicals. This is achieved using a tetrazolium salt as a superoxide detector and adding xanthine oxidase. The assay was performed according to the manufacturer’s guidelines (Assay kit, Cayman Chemical, Ann Arbor, MI). In short, 10 µl of standards and test samples (2 µg protein each) were diluted in 200 µl of tetrazolium salt solution, prepared in duplicate. The reaction was initiated by adding 20 µl of xanthine oxidase solution to each well, followed by a 30-minute incubation. Absorbance was read at 450 nm using a Tecan Infinite M200 plate reader. The assay’s sensitivity was 0.005 U/ml of SOD activity, with intra- and inter-assay coefficients of variation (CVs) of 2.5% and 15%, respectively.

*Glutathione peroxidase (GPx)* activity eliminates hydrogen peroxide by using reduced glutathione (GSH) as a cofactor, producing oxidized glutathione (GSSG). GSSG is then converted back to GSH through the action of glutathione reductase (GRed), which requires NADPH. Thus, when GSH and GRed are present in excess, the rate of NADPH consumption directly reflects GPx activity. A standard curve was generated using varying concentrations of NADPH, each tested in duplicate. Test samples (3.5 µg protein) were combined in triplicate with 200 µl phosphate buffer (100 mM, pH 7.5) containing EDTA (0.6 mM), NADPH (0.25 mM), GSH (2.5 mM), GRed (0.84 U/ml; Fluka), and tert-butyl hydroperoxide (TBHP, 0.8 mM; Fluka) in excess. Absorbance at 340 nm was recorded every minute over 5 min using a Tecan Infinite M200. GPx activity was expressed in nmol/min/µl, with a detection limit of 0.05 nmol/min/µl. The intra- and inter-assay CVs were 2.5% and 14%, respectively.

*Glutathione reductase (GRed)* activity was determined in the same way as GPx, using the identical NADPH standard curve. Here, NADPH consumption was measured in a solution containing 200 µl phosphate buffer (100 mM, pH 7.5), EDTA (0.6 mM), oxidized glutathione (GSSG, 2.5 mM), and NADPH (0.25 mM), with each reaction run in triplicate (3.5 µg protein per sample). Absorbance at 340 nm was measured every minute for 5 min, and GRed activity was expressed in nmol/min/µl, representing the amount of NADPH used to reduce GSSG. The assay sensitivity was 0.05 nmol/min/µl, with intra- and inter-assay CVs of 2.3% and 17%, respectively.

For all biomarkers, any value below the assay detection limit was recorded as zero. The *GPx/GRed ratio* was calculated to reflect the interplay between the two enzymes in maintaining cellular redox balance (Alameda et al., 2018; Schilliger et al., 2024).

### 2.5. Statistical analyses

Statistical analyses were conducted using R (v4.3.2) and RStudio (v2025.09.1). Figures were generated using ggplot2 (Wickham et al., 2016). To prevent bias, one extreme outlier in GRed (residual >2.5) was removed from the statistical analysis. Consequently, the sample size was 115 participants for the analysis of GRed and 116 participants for GPx and SOD.

Four conditional random forest regressions were performed to predict OS biomarkers (SOD, GPx, GRed, GPx/GRed) in young adulthood using global CM measures (DURATION, SEVERITY, MULTI) and age-specific CM categories (ABUSE, NEGLECT, PEER, SEXA) as predictors. To minimize potential sources of bias, we controlled for covariates that could influence OS levels. Specifically, we adjusted for age at blood draw, sex (male/female), BMI, fasting status at the time of blood collection (yes/no), smoking (yes/no), medication use (yes/no), the time interval between blood collection and laboratory processing, substance use during the past 30 days (cannabis, cocaine, amphetamines, opiates; each yes/no), and risky alcohol use (yes/no). CM types with a prevalence of less than 5% at a given age were excluded. This concerned sexual abuse between 3–13 and 15–18 years and peer violence between 3 and 6 years.

Unlike linear regression, which assumes normally distributed residuals and is sensitive to multicollinearity, conditional random forest can manage high predictor collinearity and do not rely on distributional assumptions (Breiman, 2001). This machine learning approach constructs multiple decision trees using random data subsets, mitigating overfitting through a 75% training and 25% testing split. Variable importance (VI) was determined by permuting predictors, refitting the model, and assessing changes in mean square error (MSE). Greater MSE increases indicated higher predictor importance. VI estimates were averaged over 100 permutations, and significance was assessed using 5,000 iterations with reshuffled outcomes, applying Z-tests to identify significant predictors (Schalinski & Teicher, 2015). Because VI only provides information on the relative importance of predictors, but not on the *direction* of the relationship between biomarkers and predictors (Juen et al., 2024), Spearman correlation coefficients or t-tests were calculated between the most important predictors identified in the conditional random forest regression models and the OS biomarkers.

## 3. Results

### 3.1. Sample characteristics

Participants (*N* = 116) were on average 26.29 years old (*SD* = 3.52) and 31% were women. On average, the first out-of-home placement occurred at the age of 11.4 years. Overall, 90.1% reported exposure to at least one form of CM above previously established threshold (Teicher & Parigger, 2015). See Table 1 for more information.

**Table 1.** Description of the sample (*N* = 116).

Variable	*M* (*SD*)
Female sex (*N* [%])	35 (31%)
Age	26.29 (3.59)
Prevalence of current diagnosis (SKID-I and II) (*N* [%])	99 (85%)
Substance use disorders	51 (44%)
Any personality disorders	48 (41%)
ADHD	29 (25%)
Mood disorders	28 (24%)
Anxiety disorders	18 (16%)
PTSD	6 (5%)
Psychotic disorders	3 (3%)
Currently taking medication (*N* [%])	48 (43.6%)
Substance use in the last 30 days (*N* [%])	
Cannabis	54 (47%)
Cocaine	16 (14%)
Amphetamine	7 (6%)
Opiate	3 (3%)
Smokers, n (%)	78 (70%)
Risky alcohol consumption (AUDIT>7) (*N* [%])	39 (34%)
Number of placements	4.11 (4.07); range [1; 20]
Level of Oxidative Stress	
SOD (U/ml/ug prot)	0.17 (0.07)
GRed (nmol/min/ug)	0.01 (0.01)
GPx (nmol/min/ug)	0.06 (0.02)
GPx/GRed ratio	5.05 (2.79)
Childhood maltreatment (MACE-X)	
Multiplicity (MULTI)	3.16 (2.39)
Severity (SEVERITY)	31.69 (17.18)
Duration (years) of overall CM (DURATION)	10.13 (7.34)
BMI (in kg/m^2^)	24.88 (5.00)
Fasted before blood collection (*N* [%])	45 (47%)
Time in minutes between blood collection and laboratory processing	17.50 (18.17)

Note: SCID = Structured Clinical Interview for DSM-IV, AUDIT = Alcohol Use Disorders Identification Test, SOD = superoxide dismutase, GPx = glutathione peroxidase, GRed = glutathione reductase, CM = childhood maltreatment, BMI = body mass index.

**Figure 1.**
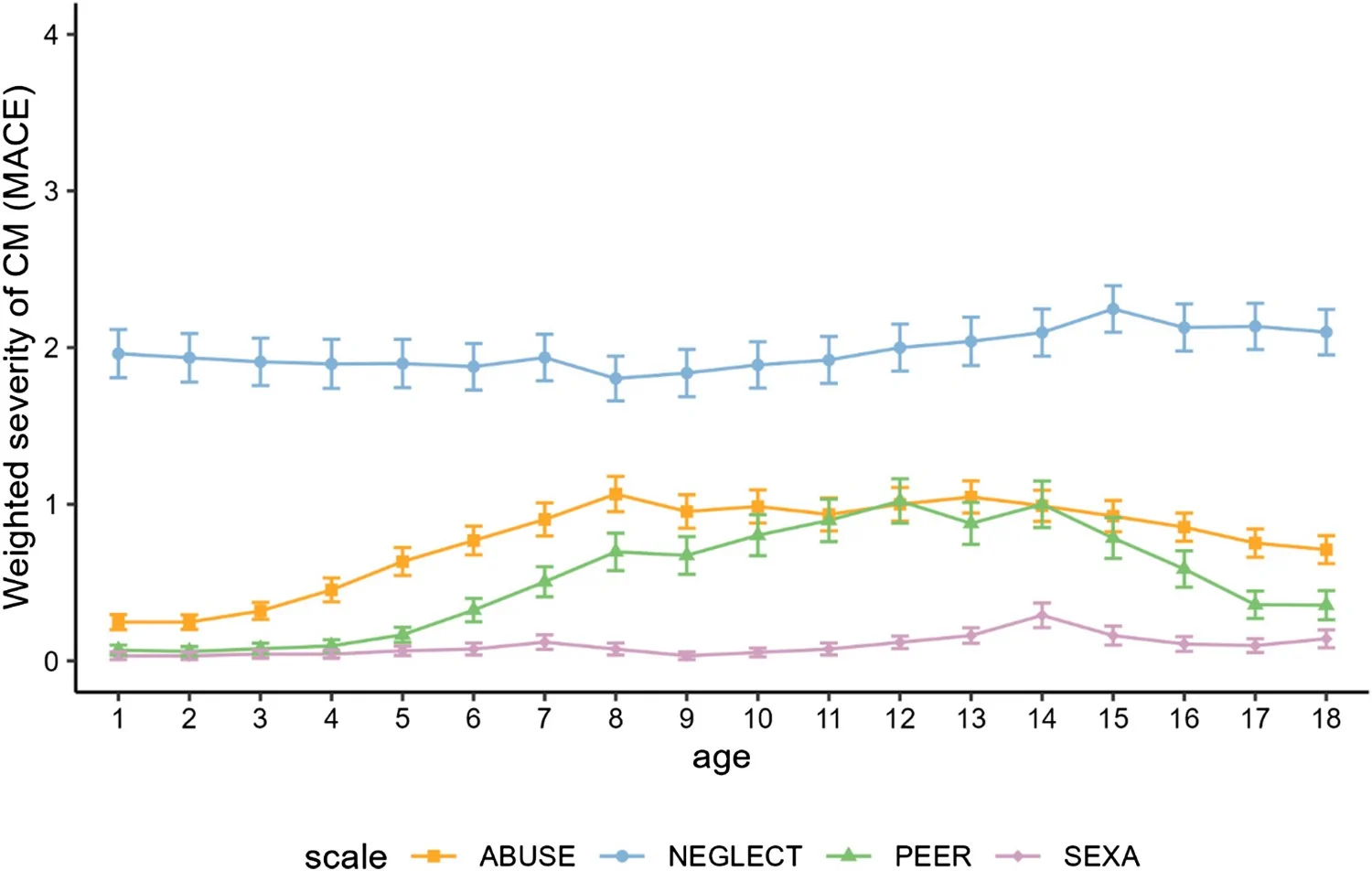
Weighted severity scores (0–10) per year of life for parental abuse (ABUSE), neglect (NEGLECT), peer violence (PEER), and sexual abuse (SEXA). Error bars indicate standard errors (SE). Note: The severity of each CM type per year of life was quantified using a weighted score ranging from 0 to 10 to ensure comparability across the four CM types. MACE = Maltreatment and Abuse Chronology of Exposure.

Figure 1 illustrates the mean severity of each type of CM across the first 18 years of life. Neglect emerged as the most prevalent form, with consistently elevated levels throughout childhood and adolescence. Parental abuse showed an initial increase from approximately age 3, reaching peaks at ages 8 and 13, followed by a modest decline after age 14. Peer violence began to rise around age 5, with pronounced peaks at ages 12 and 14, and subsequently declined. Sexual abuse was reported least frequently among all forms of CM.

### 3.2. Predicting OS from CM types, timing and duration

**Figure 2.**
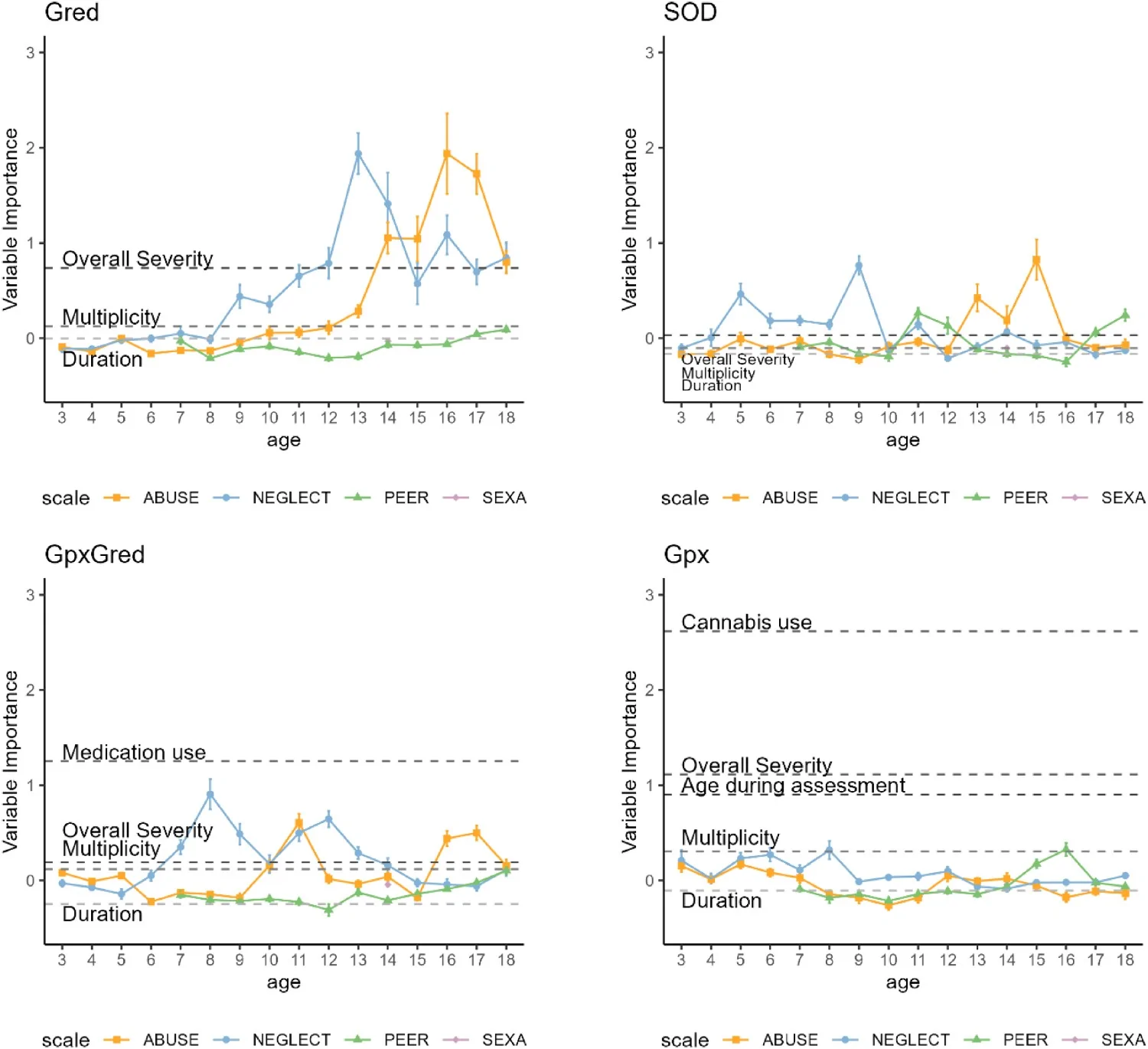
Variable importance of type- and timing-specific characteristics of CM and the global multiplicity, duration, and severity on the oxidative stress biomarkers. Note: The dashed lines show the variable weighting for the global multiplicity, duration, and severity and of the covariates with a high variable importance (VI).

Conditional random forest regression models indicated that parental neglect and abuse in specific ages were predictors with high VI to predict SOD, GRed and GPx/GRed (cf. Figure 2). Specifically, for SOD, parental abuse at age 15 (*VI* = 0.83, *p* = .041) and neglect at age 9 (*VI* = 0.76, *p* = .033) were found to exhibit the highest VI. Overall severity, the multiplicity of different CM types, the duration of CM, and the covariates were exhibited lower and non-significant VI in this model. The follow-up analyses did not reveal a significant correlation between SOD and abuse at the age 15 (*rs* = −0.18, *p* = .055) and neglect at age 9 (*rs* = −0.12, *p* = .193; cf. Table 2).

**Table 2.** Spearman correlation between oxidative stress biomarkers and CM types in important ages.

OS biomarker	CM type (age)	*n*	*rs*	*p*-value
SOD	Abuse (15)	116	−.18	.055
	Neglect (9)	116	−.12	.193
GPx	CM total score	115	.03	.774
	age	115	−.11	.222
GPx/GRed	Neglect (8)	115	.26	.005**
	Neglect (12)	115	.26	.005**
GRed	Abuse (14)	115	.20	.034*
	Abuse (15)	115	.19	.038*
	Abuse (16)	115	.22	.019*
	Abuse (17)	115	.20	.033*
	Abuse (18)	115	.13	.157
	Neglect (13)	115	−.23	.015*
	Neglect (14)	115	−.20	.034*
	Neglect (16)	115	−.18	.056
	Neglect (17)	115	−.18	.050
	Neglect (18)	115	−.20	.034*

Note: CM = childhood maltreatment, * *p* < .05; ** *p* < .01.

For GRed, the predictors with the highest VI were parental abuse between the ages of 14 and 18 (highest VI at age 16, *VI* = 1.94, *p* = .005). Furthermore, neglect between the ages of 13 and 18 (except at age 15) exhibited a high VI in the model predicting GRed (highest VI at age 13, *VI* = 1.94, *p* = .003). Overall severity, duration, multiplicity, and the covariates were not identified as predictors with significant VI for GRed. The follow-up analyses revealed significant negative correlations between GRed and neglect at ages 13 (*rs* = −.23, *p* = .015), 14 (*rs* = −.20, *p* = .034), 16 (*rs* = −.18, *p* = .056), 17 (*rs* = −.18, *p* = .050), and 18 (*rs* = −.20, *p* = .034). Furthermore, there was a significantly positive association between GRed and parental abuse at ages 14 (*rs* = .20, *p* = .034), 15 (*rs* = .19, *p* = .038), 16 (*rs* = .22, *p* = .019), 17 (*rs* = .20, *p* = .033), and 18 (*rs* = .13, *p* = .157).

For the GPx/GRed ratio, medication use (*VI* = 1.25, *p* = .018) was showed the highest VI, followed by neglect at the age of 8 (*VI* = 0.91, *p* = .025) and 12 (*VI* = 0.64, *p* = .049). The VI of overall severity, duration, multiplicity, and the covariates were not identified as significant in the random forest model predicting GPx/GRed. In the follow-up analyses, a higher GPx/GRed ratio was associated with significantly more severe neglect at age 8 (*rs* = .26, *p* = .005) and 12 (*rs* = .26, *p* = .005). Furthermore, the GPx/GRed ratio was significantly higher among individuals using medication (*M* = 5.82, *SD* = 3.09) compared with those not using medication (*M* = 4.46, *SD* = 2.44), *t*(107) = −2.56, *p* = .012.

For GPx, cannabis use was the predictor with the highest VI (*VI* = 2.62, *p* = .005), followed by overall CM severity (*VI* = 1.11, *p* = .025) and age during assessment (*VI* = 0.90, *p* = .049), while the CM categories, timing, duration, multiplicity, and the covariates exhibited non-significant VIs. In the follow-up analyses, individuals who used cannabis exhibited significantly lower GPx levels (*M* = 0.05, *SD* = 0.02) compared with those who did not use cannabis (*M* = 0.06, *SD* = 0.02), *t*(113) = 2.93, *p* = .004. There was no significant association between GPx and total CM severity (*rs* = .03, *p* = .774), and age at assessment (*rs* = −0.11, *p* = .222). Spearman correlation plots are provided in the Supplementary Material (Figure S2).

## 4. Discussion

This study explored the associations between the types, timing, duration, multiplicity and overall severity of CM with OS biomarkers in young adults who experienced out-of-home placements. To our knowledge, this is the first study investigating the differential impact of CM types and timing on OS. The findings suggest that CM during certain periods was associated with OS in young adulthood. Specifically, abuse during adolescence was associated with increased GRed levels, whereas neglect during adolescence was associated with decreased GRed levels. Neglect during middle childhood and medication use was associated with an increased GPx/GRed ratio. Abuse during adolescence and neglect during middle childhood emerged as the predictors with the highest VI for SOD; however, these associations could not be confirmed in follow-up regression analyses. We found no evidence of an association between GPx and CM type and timing, but it was decreased in participants with cannabis use.

The results suggest that CM exposure at specific developmental stages is associated with differences in OS biomarkers activity, particularly GRed. Specifically, parental abuse between the ages of 14 and 17 was related to increased GRed activity, whereas neglect occurring between the ages of 13 and 18 (except 15) was associated with reduced GRed activity. Furthermore, the GPx/GRed ratio was increased following neglect at the age 8 and 12 and it was higher among individuals taking medication, compared with those not taking medication. These findings suggest that the redox system is developmentally sensitive and that CM type and timing may be linked to long-term changes in OS regulation – a finding that should be replicated in larger samples given the limited statistical power of the current work.

Previous research highlights prepuberty as a vulnerable period during which CM can have lasting effects on behaviour and mental health. CM in infancy is linked to impaired neurocognitive functioning (Cowell et al., 2015), while early parental abuse predicts later psychopathology (Meier, Gurri, et al., 2025). A meta-analysis further identified middle childhood (6–13 years) as the highest-risk period for depression following CM (Li et al., 2023). On the biological level, CM has been associated with altered DNA methylation (Lussier et al., 2023), disrupted mitochondrial function (Mposhi & Turner, 2023), increased inflammation in girls exposed before age 5 (Ehrlich et al., 2021), and sex-specific effects on pubertal timing (Negriff et al., 2015). In contrast, our study did not identify prepuberty as particularly vulnerable period for alterations in OS biomarkers. One possible explanation is the long interval between prepubertal CM exposure and biomarker assessment, during which effects may have been diluted by later experiences and interventions.

This study identified a small-to-moderate association between abuse in mid- to late adolescence and altered antioxidant levels in young adulthood. Prior findings on puberty and adolescence as vulnerable periods for CM are mixed: maltreatment during early childhood is more often linked to internalizing symptoms, whereas adolescent exposure has been associated with broader psychosocial difficulties (Thornberry et al., 2010). Neurobiological and physical health outcomes have also been linked to CM across both pre- and post-pubertal phases, including heightened amygdala reactivity (Sicorello et al., 2021) and increased risk of somatic disease (Riem & Karreman, 2019). Our results add to this body of evidence by suggesting that adolescence may represent a window of vulnerability for OS in adulthood.

Our study identified distinct vulnerable periods for different maltreatment types: neglect in middle childhood was linked to reduced GRed level, whereas parental abuse in adolescence was associated with increased GRed levels in young adulthood, which suggests that both the timing and the type of CM contribute to its long-term consequences. This is in line with previous research showing that neglect in early and middle childhood was associated with neurobiological changes, such as structural changes in the amygdala and hippocampus (Herzog et al., 2020), reduced grey matter volume (Fujisawa et al., 2018), and hypothalamic-pituitary-adrenal (HPA) axis dysregulation as evident in decreased adult hair cortisol (Schalinski et al., 2019). Beyond those alterations on the neurobiological level, physical and emotional neglect in early childhood were associated with increased symptoms of dissociation, whereas emotional neglect in middle childhood was linked to increased symptoms of depression (Schalinski et al., 2016). Together, these findings suggest that neglect, particularly during early developmental windows of heightened neuroplasticity, may contribute to long-term neurobiological vulnerabilities and increased risks of mental health disorders (Miskolczi et al., 2019). Abuse, on the other hand, has previously been associated with externalizing behaviours and aggression when occurring in infancy and early childhood (Manly et al., 2001). Early-life abuse can impact cognitive functions such as attention, learning, and memory (Schalinski et al., 2018). It has been linked to altered immune function (Slopen et al., 2013) and a heightened risk of depression later in life (Dunn et al., 2013). It is possible that OS is an underlying mechanism that contributes to the observed alterations, i.e. changes in immunity, psychiatric disorders, or brain alterations. In sum, these findings underscore the lasting impact of both neglect and abuse, emphasizing the need for early identification and intervention strategies tailored to the developmental stage and type of maltreatment.

For GPx activity, the most important predictor was cannabis use, while the characteristics of CM played a less prominent role in this context. However, when examining the GPx/GRed ratio, an indicator of the GSH cycle, were identified, in that neglect during middle childhood was associated with an increased GPx/GRed ratio, suggesting increased activity of the GSH cycle. This indicates the importance of assessing interrelated OS biomarkers rather than isolated measures when they are reflective of a coordinates process. Moreover, this complexity suggests that failing to consider CM types and timing in OS studies may lead to null findings due to complex interactions among OS regualtors.

In our sample, GPx was reduced in participants who reported cannabis use within the last 30 days. This finding is consistent with prior evidence demonstrating the antioxidant properties of cannabis and its components (Graczyk et al., 2021; Kopustinskiene et al., 2022). In addition, we observed an association between the GPx/GRed ratio and medication use. Consistent with the literature (Lee et al., 2013; Miniksar et al., 2023), the GSH cycle was activated in those using medication. Due to the high heterogeneity of the types and doses of medications used and small sample sizes, we could not follow up upon this finding with more fine-grained analyses. Nevertheless, these results highlight that medication use should be taken into account when investigating OS in order to control for possible confounding effects.

### 4.1. Limitations and strengths

The present findings should be interpreted in light of some limitations. Random forest regression models offer important advantages: they can handle missing data effectively (Qi, 2012), are robust to multicollinearity (Strobl et al., 2007), and perform well in detecting complex, non-linear relationships (Qiu et al., 2010; Wager & Athey, 2018; Wang et al., 2016). They are also useful for narrowing down a large set of candidate variables to a smaller group of potentially relevant predictors (Boulesteix et al., 2012). However, the current study was based on a relatively small sample size, particularly given the number of predictors included. Although random forest regression models are generally more robust in small samples than traditional regression models, their performance and stability may still be affected by the limited sample size (Luan et al., 2020). In particular, insufficient sample size may compromise model reliability, as indicated by unstable out-of-bag error estimates (Fife & D’Onofrio, 2023). Furthermore, no correction for multiple testing was applied to the results. The rationale for this decision is the exploratory nature of the study. Importantly, the absence of a significant effect in the random forest regression model should not be interpreted as evidence that a predictor is unrelated to the outcome. Rather, random forest regression estimates reflect the relative contribution of predictors within a multivariable framework that accounts for shared variance among variables. Consequently, predictors that do not emerge as significant in the forest regression models may still be associated with the outcome and remain of substantial theoretical and clinical relevance. Therefore, the present findings should be considered exploratory and hypothesis-generating, and a replication in larger, independent samples is warranted.

Second, OS biomarkers were measured only once, in young adulthood. It is conceivable that CM induces short-term or dynamic changes in OS that are no longer detectable many years after the exposure. A second measurement point during childhood or adolescence would have been valuable to provide a more comprehensive understanding of the temporal relationship between CM and OS. In addition, a third assessment of OS biomarkers in participants’ 40s or 50s would be valuable for examining the long-term effects of CM type and timing on OS biomarkers.

Third, the blood sampling protocol was not optimized for assessing OS markers, as there were variations in the time between blood draw and laboratory processing that could introduce measurement variability or bias (Murphy et al., 2022). Additionally, inconsistencies in fasting status and the high proportion of smokers in the sample may have introduced noise in the biomarker data. Although participants were advised to fast and samples were placed on ice and processed shortly after interviews, full adherence to these protocols could not always be ensured. While we statistically controlled for the fasting status and delay between blood collection and laboratory processing, residual confounding cannot be ruled out.

Fourth, the sample was composed predominantly of individuals with a history of out-of-home-placement and CM. This limits the generalizability of the findings. Future research should aim to include a representative control group without previous out-of-home placement to allow for comparisons and better account for potential distributional biases. In addition, even though the MACE-X assesses 10 different types of CM, the subscales were aggregated into four broader categories, i.e. parental abuse, parental neglect, peer victimization and sexual abuse. This aggregation may have obscured small effects of individual subtypes. However, inclusion of all ten individual subtypes was not feasible, as it would have required incorporating an excessive number of predictors into the models, thereby rendering the estimation of VI less precise and more challenging. Importantly, the prevalence of sexual abuse was below 5% in most years of upbringing. We therefore excluded the years with very low prevalence of sexual abuse from the analyses. Consequently, no conclusions can be drawn regarding the association between sexual abuse in early to middle childhood and OS. Further research is required to address this question.

Finally, all self-report measures – including CM exposure – may be subject to biases such as social desirability, limited self-awareness (Kessler et al., 2000), and recall bias (Esser et al., 2002; Reuben et al., 2016). However, self-reports remain widely used and accepted in psychological research, and there is evidence suggesting self-reports may capture subjective aspects of maltreatment more relevant to adult mental health than official records (Danese & Widom, 2020).

Despite these limitations, this study took benefit from a highly vulnerable population with documented cases of CM. This population is rarely represented in empirical research due to its limited accessibility, making the sample particularly unique and informative of a highly vulnerable at-risk population. In addition, the study employed conditional random forest regression models – a machine learning approach capable of handling multicollinearity and complex data structures. This allowed for a more nuanced examination of how specific characteristics of CM, especially type and timing, are linked to OS. Importantly, the findings contribute to the limited but growing body of research on OS as a biological mechanism underlying the long-term effects of CM. The results provide preliminary insights into the complex interplay between CM and OS and underscore the need for further investigation of this relationship, which has so far received relatively little empirical attention. Given the exploratory nature of the present study, the findings should be interpreted with caution and viewed as a basis for future research rather than as conclusive evidence.

### 4.2. Implications

Targeted interventions to rebalance the OS system may be important for young adults with a history of CM (Preiser, 2012). Established strategies include a nutrient-rich diet (Aikawa et al., 2002; Ilari et al., 2025; Qiu et al., 2010), healthy sleep routines (Everson et al., 2005) and regular physical activity, particularly moderate aerobic exercise (Camiletti-Moirón et al., 2013; Poljsak, 2011; Zalavras et al., 2015) and may offer a promising, accessible avenue for reducing OS in individuals exposed to CM. These approaches are particularly relevant for vulnerable populations such as children and adolescents in youth welfare institutions. Given the difficulty of reducing established OS imbalance (Poljsak, 2011), preventive strategies – preventing CM, early detection, and timely intervention – are particularly important. Clinical care for CM survivors should integrate trauma-informed psychotherapeutic support with interventions considering types and timing of CM beyond its duration, multiplicity and severity. A multidimensional approach combining prevention, early intervention, and support in building a healthy lifestyle may be key to mitigating the long-term consequences of CM.

The use of conditional random forest regression models indicated that considering both, the type and timing of exposure may be important to evaluate association between CM and neurobiological alterations. Furthermore, the potential involvement of OS as an underlying mechanism highlights the need for further research investigating its interaction with other biological systems, such as the HPA axis and inflammatory pathways, in order to advance a more comprehensive understanding of the biological consequences of CM.

## 5. Conclusion

This exploratory study examined associations between type and timing of retrospectively assessed CM and OS in young adulthood. The findings provide preliminary evidence that types and timing of CM may be differentially associated with OS. While these results should be interpreted with caution and require replication in larger and longitudinal studies, they underscore the relevance of considering both the type and timing of CM in future research. A better understanding of these associations may ultimately contribute to the identification of vulnerable developmental periods and inform prevention and intervention strategies.

## Supplementary Material

Supplemental Material

## Data Availability

Data is available upon reasonable request.
