# Supplementary material for: Associations of childhood maltreatment types and timing with oxidative stress in formerly out-of-home placed young adults
Source: Eur J Psychotraumatol. 2026 Sep 2;17(1):2711481. doi: 10.1080/20008066.2026.2711481 (PMC13539789; doi:10.1080/20008066.2026.2711481)
Supplement: Supplementary file 1 [file ZEPT_A_2711481_SM7424.docx]

# Supplemental material

**Figure S1**

Flow Chart of Study Participants from baseline (MAZ. study) to follow-up (JAEL study) with final sample sizes included in data analyses.

**Follow-Up (JAEL, t2)**

**Follow-Up (JAELred, t2)**

Reason for drop-out (*n*=15):

- No valid measurement of OS biomarkers (n=15)
- Outlier in GRed (n=1)

Reason for drop-out (n=55):

- Afraid of needles (n=4)
- Digital Assessments (n=8)
- Weekend Assessments/ lab not open (n=7)
- Did not want to (n=13)
- Other (n=23)

Reason for drop-out (*n*=46):

- Unable to schedule a meeting and motivate participants to come to the in-person interviews (n=46)

Participants with available blood sample for follow-up study JAELred (N=131)

Face-to-Face Interviews of JAEL (*N*=185)

Included in the analytical sample (*N*=116)

(for GRed *N*=115)

Reason for drop-out (*n*=81):

- Did not consent to be contacted for follow-up (*n=*81)

Participants with consent for follow-up (*N*=511)

**Baseline (MAZ., t1)**

Included in the baseline MAZ. study (*N*=592)

Included in the follow-up study JAEL (*N*=231)

Reason for drop-out (*n*=280):

- Refused to participate (n=99)
- Did not provide informed consent (*n*=44)
- Could not be reached (*n*=129)
- Deceased (*n*=8)

**Figure S2**

Spearman's rank correlation coefficient between OS biomarkers and significant predictors from the random forest regression model

|  |  |  |
| --- | --- | --- |
|  |  |  |
|  |  |  |
|  |  |  |
|  |  |  |
|  |  |  |
|  |  |  |

Note. The age at which the CM types were experienced is given in brackets.
